# A Study on the Clinical Factors Associated with Acute Appendicitis and Perforated Appendicitis among Children in a Secondary Medical Centre in Malaysia

**DOI:** 10.21315/mjms2020.27.4.13

**Published:** 2020-08-19

**Authors:** Pheng Hian Tan, Xiu Xin Teng, Zhen Yao Gan, Siew Qin Tan

**Affiliations:** 1 Department of Surgery, Hospital Seberang Jaya, Seberang Jaya, Pulau Pinang, Malaysia

**Keywords:** appendicitis, diagnosis, risk, rupture, child

## Abstract

**Background:**

Appendicitis complicated with appendiceal perforation is common among children. The delay in diagnosis of appendicitis is due to children’s varied presentations and their difficulty in communicating symptoms. We aimed to identify clinical factors that aid in predicting acute appendicitis (AA) and perforated appendicitis (PA) among children.

**Methods:**

This retrospective study involved 215 children aged 12 years and below with the initial diagnosis of AA and PA. Clinical factors studied were demographics, presenting symptoms, body temperature on admission (BTOA), white cell count (WCC), absolute neutrophil count (ANC), platelet count and urinalysis. Simple and multiple logistic regressions were used to determine the odds ratio of the statistically significant clinical factors. Results: The mean age of the included children was 7.98 ± 2.37 years. The odds of AA increased by 2.177 times when the age was ≥ 8 years (*P* = 0.022), 2.380 times when duration of symptoms ≥ 2 days (*P* = 0.011), 2.447 times with right iliac fossa (RIF) pain (*P* = 0.007), 2.268 times when BTOA ≥ 38 °C (*P* = 0.020) and 2.382 times when neutrophil percentage was ≥ 76% (*P* = 0.045). It decreased by 0.409 times with non-RIF pain (*P* = 0.007). The odds of PA was increased by 4.672 times when duration of symptoms ≥ 2 days (*P* = 0.005), 3.611 times when BTOA ≥ 38 °C (*P* = 0.015) and 3.678 times when neutrophil percentage ≥ 76% (*P* = 0.016). There was no significant correlation between WCC and ANC with AA and PA.

**Conclusion:**

Older children with longer duration of symptoms, RIF pain and higher BTOA are more likely to have appendicitis. The risk of appendiceal perforation increases with longer duration of symptoms and higher BTOA.

## Introduction

Appendicitis is the most common paediatric surgical emergency. It will progress to appendiceal perforation if treatment is delayed, leading to intrabdominal abscess, peritonitis and a higher risk of postsurgical complications such as paralytic ileus. The appendiceal perforation rate (APR) is high among children, ranging from 30% to 74% (1–3). The delay in diagnosis is due to children’s varied presentations and their difficulty in communicating symptoms (4–6). Clinical factors such as the duration of symptoms, white cell count (WCC), absolute neutrophil count (ANC) and C-reactive protein (CRP) have been shown to correlate with the risk of appendiceal perforation (7–13). Studies in the United States even show that health insurance status correlates with the risk of appendiceal perforation (14–16). Modern imaging modalities such as ultrasound and computed tomography (CT) (17–19) are useful in diagnosing appendicitis and its complications, yet they are not easily accessible. Thus, it is imperative for clinicians to diagnose acute appendicitis (AA) and perforated appendicitis (PA) accurately by clinical examination and basic laboratory investigation such as full blood count. However, to date, there is no consensus on the factors that predict AA and PA in children. Thus, we seek to identify the clinical and biochemical factors that correlate with AA and PA in children to improve our diagnostic accuracy.

## Methods

This is a retrospective study carried out in Hospital Seberang Jaya, a secondary medical centre in Penang, Malaysia. All paediatric patients aged less than 12 years old who were first diagnosed with AA by the surgical team from 1st January 2016 to 31st December 2018 were recruited. Exclusion criteria were patients whose parents opted for discharge at own risk and patients with significant comorbidities who were transferred to tertiary hospitals. A total of 215 patients were available for study. All appendicectomies were conducted by senior medical officers in the surgical department using the open approach. All appendix specimens were sent for histopathological examination (HPE).

The objective of this study is to identify the clinical factors associated with true appendicitis and PA. For the purpose of this study, the diagnoses of normal appendix, non-perforated appendicitis and PA were made based on HPE findings. The possible clinical factors analysed include age, gender, presenting symptoms, body temperature on admission, WCC, platelet count and urinalysis.

All data were analysed using SPSS software version 22 (Chicago, Illinois, USA). Simple logistic regression was run for each possible clinical factor, and multiple logistic regression was subsequently run for clinical factors with statistical significance. Statistical significance was set at *P* < 0.05.

## Results

The mean age of the sample was 7.98 ± 2.37, and 60.5% of patients were male. Malays constituted 60.5%, Indians constituted 28.8%, whereas Chinese constituted 9.8%. About 46.0% patients had AA, and among them, 49.5% had PA. The mean duration of symptoms was 2.40 ± 2.11 days. All patients presented with abdominal pain, with 53.0% having right iliac fossa (RIF) pain and 47.0% having non-RIF abdominal pain. About 74.0% patients had vomiting, 66.5% had fever, 27.9% had diarrhoea, 16.3% had symptoms of upper respiratory tract infection and only 8.4% had dysuria. The mean body temperature on admission was 37.98 ± 0.81 °C, and the mean duration of fever was 2.13 ± 2.14 days. The mean WCC was 15.21 ± 5.78 × 10^3^/μL, and the mean platelet count was 341.02 ± 85.16 × 10^3^/μL. The mean neutrophil percentage was 72.54 ± 14.74% and the mean ANC was 11.76 ± 5.95 × 10^3^/μL. Only 54.9% of our study population had urinalysis, and among them, 36.4% had urine ketone, 9.3% had urine leukocyte and 2.5% had urine nitrite. The mean duration of admission was 2.52 ± 2.15 days and the mean duration of surgery was 63.29 ± 25.55 min.

The factors independently associated with AA with statistical significance were age ≥ 8 years; duration of symptoms ≥ 2 days; presenting symptoms such as RIF pain, non-RIF abdominal pain, nausea and vomiting, fever and dysuria; body temperature on admission ≥ 38 °C; WCC ≥ 15 × 10^3^/μL, neutrophil percentage ≥ 76% and ANC ≥ 12 × 10^3^/μL, as shown in Table 2. In multiple logistic regression, as shown in Table 3, only age ≥ 8 years, duration of symptoms ≥ 2 days, RIF pain, non-RIF abdominal pain, body temperature on admission ≥ 38 °C and neutrophil percentage ≥ 76% demonstrated statistical significance. The odds of AA were 2.447 times greater with RIF pain (*P* = 0.007) and 0.409 times lower with non-RIF abdominal pain (*P* = 0.007). The odds of AA increased by 2.177 times for age ≥ 8 years (*P* = 0.022), 2.380 times when the duration of symptoms ≥ 2 days (*P* = 0.011), 2.268 times when the body temperature on admission ≥ 38 °C (*P* = 0.020) and 2.382 times when the neutrophil percentage ≥ 76% (*P* = 0.045).

The factors independently associated with PA with statistical significance were gender, duration of symptoms ≥ 2 days, fever, body temperature on admission ≥ 38 °C and neutrophil percentage ≥ 76%, as shown in Table 4. In multiple logistic regression, as shown in Table 5, only duration of symptoms ≥ 2 days, body temperature on admission ≥ 38 °C and neutrophil percentage ≥ 76% demonstrated statistical significance. The odds of PA increased by 4.672 times when the duration of symptoms ≥ 2 days (*P* = 0.005), 3.611 times when the body temperature on admission ≥ 38 °C (*P* = 0.015) and 3.678 times when the neutrophil percentage ≥ 76% (*P* = 0.016).

## Discussion

Appendicitis in children is commonly missed and its prevalence ranged from 3.8%–28.0% (20–22). This is attributable to children’s varied presentations. Becker et al. (4) reported that 44% out of 755 patients presented with > 6 atypical features including absence of symptoms such as fever, migration of abdominal pain, nausea and vomiting and absence of signs such as tenderness at RIF and signs of local peritonitis. Appendicitis is commonly misdiagnosed as gastroenteritis. Cappendijk and Hazebroek (5) reported that 38 out of 78 patients (48.7%) who were diagnosed as appendicitis 48 h after onset of symptoms presented with diarrhoea and 32 (41.0%) were initially diagnosed as gastroenteritis. Thus, patients with progressive abdominal pain or deterioration in apparent or confirmed gastroenteritis are indicated for reassessment by surgeon to rule out appendicitis, as suggested by Murch (23). Gardikis et al. (6) reported that patients may present with only urological symptoms such as right renal colic, dysuria, frequency and urinary retention. This is due to the proximity of appendix to the right distal ureter and urinary bladder. Similar to previous studies, there were 28.7% and 8.4% of our study population, respectively, who presented with diarrhoea and dysuria, and this posed a possibility for misdiagnosis (Table 1). Delay in diagnosis of appendicitis may lead to PA. APR stands at 24% if appendicitis is diagnosed in < 48 h from symptom onset. It increases to 71% if diagnosis is made > 48 h from symptom onset (5). It is, thus, evident that a timely diagnosis of AA and PA is important.

The significance of abdominal pain and its location is questionable in paediatric population as younger children may not convey their symptoms accurately. Ngim et al. (7) reported that both RIF pain and non-RIF abdominal pain were observed in similar fashion in children with and without appendicitis. In contrary, our study showed that the odds of AA are significantly increased with RIF pain and decreased with non-RIF abdominal pain (Table 3). However, the symptoms were not significant in differentiating AA and PA (Table 4).

Clinical factors that correlated with PA include age, duration of symptoms, temperature on admission, WCC and CRP. Bansal et al. (24) reported that APR is directly related to patients’ age, whereby APR stands at 86% and 60% among children aged < 1 year and < 5 years, respectively. Our study showed that the odds of AA is higher for children aged ≥ 8 years (Table 3), but age is not a significant factor in differentiating AA and PA (Table 4). Narsule et al. (10) reported that the risk of appendiceal perforation linearly increases with the duration of symptoms. APR stands at 10%, 44% and > 40% if appendicitis is diagnosed at 18 h, 36 h and > 48 h, respectively, from symptom onset. In comparison, Poudel and Bhandari (8) reported that patients with abdominal pain of > 72 h are more likely to have complicated appendicitis. Williams and Kapila (25) reported that fever is present in up to 90% of cases of appendicitis, and Siddique et al. (9) reported that body temperature on admission is higher for patients with PA. Our study showed similar results. The odds of PA increased when the duration of symptoms was ≥ 2 days and the body temperature on admission was ≥ 38°C (Table 5). However, our study showed no significant correlation between WCC and ANC with AA and PA (Table 4). Similar findings were reported by Nance et al. (11).

Numerous studies have highlighted the association of CRP with PA (9, 11, 13). We did not include CRP in our study as it is relatively costly and, thus, not routinely measured. This study is limited by our relatively small sample size. There were only 99 out of 215 (46.0%) patients with confirmed appendicitis. This is a single centre study, and this may incur selection bias as more severe cases were referred to tertiary medical centres with paediatric surgical service. Therefore, a multicentre study with larger sample size is needed to identify the factors that differentiate PA from AA.

## Conclusion

Older children with longer duration of symptoms, RIF pain and higher body temperature on admission are more likely to have appendicitis. The risk of appendiceal perforation increases with a longer duration of symptoms and a higher body temperature on admission. A combination of good clinical skills and acumens is required for making an accurate diagnosis of AA and PA in children.

## Figures and Tables

**Table 1:** Demography, presenting symptoms, full blood count and urinalysis

Age, years	7.98 ± 2.37
Male, *n* (%)	130 (60.5)
Race, *n* (%)	
Malay	130 (60.5)
Indian	62 (28.8)
Chinese	21 (9.8)
Others	2 (0.9)
Acute appendicitis, *n* (%)	99 (46.0)
Perforated appendicitis, *n* (%)^a^	49 (49.5)
Duration of symptoms, days	2.40 ± 2.11
Presenting symptoms, *n* (%)	
RIF pain	114 (53.0)
Non-RIF abdominal pain	101 (47.0)
Nausea and vomiting	159 (74.0)
Diarrhoea	60 (27.9)
Fever	143 (66.5)
Symptoms of upper respiratory tract infection	35 (16.3)
Dysuria	18 (8.4)
Temperature on admission, °C	37.98 ± 0.81
Duration of fever, days	2.13 ± 2.14
Full blood count	
White cell count, × 10^3^/μL	15.21 ± 5.78
Neutrophil percentage, %	72.54 ± 14.74
Absolute neutrophil count, × 10^3^/μL	11.76 ± 5.95
Platelet count, × 10^3^/μL	341.02 ± 85.16
Urinalysis^b^	
Urine leukocyte, *n* (%)	11 (9.3)
Urine nitrite, *n* (%)	3 (2.5)
Urine ketone, *n* (%)	43 (36.4)
Duration of admission, days	2.52 ± 2.15
Duration of surgery, min	63.29 ± 25.55

Notes:

a Only patients with AA were included, *n* = 99;

b Only 54.9% of the patients had urinalysis, *n* = 118

**Table 2:** Simple logistic regression for clinical factors possibly associated with AA

	β	SE	Wald	*P*-value	e^β^	95% CI
Age ≥ 8 years	0.659	0.283	5.426	0.020^a^	1.932	1.110, 3.363
Male	0.405	0.282	2.060	0.151	1.500	0.862, 2.609
Duration of symptoms ≥ 2 days	0.695	0.287	5.853	0.016^a^	2.003	1.141, 3.157
RIF pain	0.802	0.280	8.182	0.004^a^	2.230	1.287, 3.864
Non-RIF abdominal pain	-0.802	0.280	8.182	0.004^a^	0.448	0.259, 0.777
Nausea and vomiting	0.679	0.324	4.399	0.036^a^	1.972	1.046, 3.720
Diarrhoea	0.127	0.305	0.175	0.676	1.136	0.625, 2.064
Fever	0.975	0.306	10.160	0.001^a^	2.652	1.456, 4.829
Symptoms of URTI	−0.736	0.393	3.496	0.062	0.479	0.222, 1.036
Dysuria	1.211	0.545	4.926	0.026^a^	3.356	1.152, 9.775
Temperature on admission ≥ 38°C	0.944	0.282	11.185	0.001^a^	2.571	1.478, 4.472
WCC ≥ 15 × 10^3^/μL	0.803	0.279	8.271	0.004^a^	2.232	1.291, 3.858
Neutrophil percentage ≥ 76%	0.912	0.284	10.275	0.001^a^	2.489	1.425, 4.346
ANC ≥ 12 × 10^3^/μL	0.811	0.281	8.324	0.004^a^	2.251	1.297, 3.905
Platelet ≥ 328 × 10^3^/μL	0.211	0.274	0.591	0.442	1.234	0.721, 2.112
Urine leukocyte	−0.622	0.705	0.778	0.378	0.537	0.135, 2.138
Urine nitrite	1.135	1.240	0.838	0.360	3.111	0.274, 35.321
Urine ketone	0.284	0.389	0.534	0.465	1.329	0.620, 2.848

Notes:

a *P* < 0.050;

β = B; SE = standard error; e^β^ = adjusted odds ratio

**Table 3:** Multiple logistic regression for clinical factors independently associated with AA

	β	SE	Wald	*P*-value	e^β^	95% CI
Constant	−2.597	0.553	22.070	< 0.001	0.074	–
Age ≥ 8 years	0.778	0.339	5.253	0.022^a^	2.177	1.119, 4.234
Duration of symptoms ≥ 2 days	0.867	0.340	6.489	0.011^a^	2.380	1.221, 4.639
RIF pain	0.895	0.332	7.260	0.007^a^	2.447	1.276, 4.690
Non-RIF abdominal pain	-0.895	0.332	7.260	0.007^a^	0.409	0.213, 0.784
Nausea and vomiting	0.295	0.376	0.616	0.432	1.343	0.643, 2.803
Fever	0.648	0.370	3.062	0.080	1.911	0.925, 3.946
Dysuria	0.684	0.589	1.349	0.245	1.982	0.625, 6.289
Temperature on admission ≥ 38 °C	0.819	0.353	5.392	0.020^a^	2.268	1.136, 4.525
WCC ≥ 15 × 10^3^/μL	0.714	0.447	2.545	0.111	2.042	0.849, 4.908
Neutrophil percentage ≥ 76%	0.868	0.433	4.026	0.045^a^	2.382	1.020, 5.563
ANC ≥ 12 × 10^3^/μL	−0.359	0.549	0.429	0.512	0.698	0.238, 2.046

Notes:

a *P* < 0.050;

β = B; SE = standard error; e^β^ = adjusted odds ratio

**Table 4:** Simple logistic regression for clinical factors possibly associated with PA

	β	SE	Wald	*P*-value	e^β^	95% CI
Age ≥ 8 years	0.488	0.430	1.285	0.257	1.629	0.701, 3.785
Male	−0.948	0.438	4.676	0.031^a^	0.388	0.164, 0.915
Duration of symptoms ≥ 2 days	−1.169	0.467	6.261	0.012^a^	0.311	0.124, 0.776
RIF pain	−0.560	0.423	1.754	0.185	0.571	0.250, 1.308
Non-RIF abdominal pain	0.560	0.423	1.754	0.185	1.750	0.764, 4.006
Nausea and vomiting	0.923	0.542	2.902	0.088	2.518	0.870, 7.286
Diarrhoea	0.463	0.447	1.073	0.300	1.588	0.662, 3.812
Fever	1.216	0.531	5.244	0.022^a^	3.373	1.191, 9.547
Symptoms of URTI	−0.605	0.662	0.835	0.361	0.546	0.149, 1.999
Dysuria	−0.563	0.610	0.853	0.356	0.569	0.172, 1.881
Temperature on admission ≥ 38 °C	−1.424	0.434	10.785	0.001^a^	0.241	0.103, 0.563
WCC ≥ 15 × 10^3^/μL	−0.724	0.416	3.033	0.082	0.485	0.215, 1.095
Neutrophil percentage ≥ 76%	−1.331	0.465	8.182	0.004^a^	0.264	0.106, 0.658
ANC ≥ 12 × 10^3^/μL	−0.777	0.431	3.252	0.071	0.460	0.197, 1.070
Platelet ≥ 328 × 10^3^/μL	0.288	0.404	0.507	0.477	1.333	0.604, 2.944
Urine leukocyte	1.253	1.264	0.982	0.322	3.500	0.294, 41.702
Urine nitrite	–	–	–	–	–	–
Urine ketone	1.022	0.621	2.703	0.100	2.778	0.822, 9.389

Notes:

a *P* < 0.050;

β = B; SE = standard error; e^β^ = adjusted odds ratio

**Table 5:** Multiple logistic regression for clinical factors independently associated with PA

	β	SE	Wald	*P*-value	e^β^	95% CI
Constant	−2.852	0.935	9.303	0.002	0.058	–
Male	−0.880	0.518	2.890	0.089	0.415	0.150, 1.144
Duration of symptoms ≥ 2 days	1.542	0.547	7.938	0.005^a^	4.672	1.599, 13.563
Fever	0.887	0.620	2.047	0.153	2.428	0.720, 8.184
Temperature on admission ≥ 38 °C	1.284	0.527	5.942	0.015^a^	3.611	1.286, 10.139
Neutrophil percentage ≥ 76%	1.302	0.539	5.830	0.016^a^	3.678	1.278, 10.589

Notes:

a *P* < 0.050;

β = B; SE = standard error; e^β^= adjusted odds ratio
